# Biological adaptation in light of the Lewontin–Williams (a)symmetry

**DOI:** 10.1111/evo.14502

**Published:** 2022-05-18

**Authors:** Lutz Fromhage, Alasdair I. Houston

**Affiliations:** ^1^ Department of Biological and Environmental Science University of Jyvaskyla Jyvaskyla Finland; ^2^ School of Biological Sciences University of Bristol Bristol United Kingdom

## Abstract

Neo‐Darwinism characterizes biological adaptation as a one‐sided process, in which organisms adapt to their environment but not vice versa. This asymmetric relationship—here called Williams’ asymmetry—is called into question by Niche Construction Theory, which emphasizes that organisms and their environments often mutually affect each other. Here, we clarify that Williams’ asymmetry is specifically concerned with (quasi)‐directed modifications toward phenotypes that increase individual fitness. This directedness—which drives the adaptive fit between organism and environment—entails far more than the mere presence of cause‐effect relationships. We argue that difficulties with invoking fitness as the guiding principle of adaptive evolution are resolved with an appropriate definition of fitness and that objections against Williams’ asymmetry reflect confusions about the nature of biological adaptation.

*Adaptation is always asymmetrical; organisms adapt to their environment, never vice versa*.

George Williams, 1992

The foregoing quote by George Williams, drawn from a discussion of the “Gaia hypothesis” that Earth is a self‐regulatory system for maintaining favorable conditions for life (Lovelock 1979), provides a succinct expression of a major tenet of Neo‐Darwinism. This tenet has been called “asymmetrical externalism” (Aaby and Ramsey 2020), but here we refer to it as “Williams’ asymmetry.” Our aim in this article is to clarify what we see as misrepresentations that have led some authors to reject Williams’ asymmetry (Laland et al. 2009; Buskell 2019). The basic insight deemed to undermine Williams’ asymmetry can be traced to Lewontin (1983), who presented the following pair of differential equations to describe how standard evolutionary theory (SET) characterizes the relationship between organisms and their environment:

(1a) $$\frac{dO}{dt}=f\left(O,E\right),$$

(1b) $$\frac{dE}{dt}=g\left(E\right).$$

Here, *O* stands for organism; *E* stands for environment; *f* is a function describing how organisms evolve in response to their own and their environment's configuration, and *g* is the law of autonomous change of the environment, described as a function only of environmental variables. To describe his alternative vision, Lewontin presented the equations:

(2a) $$\frac{dO}{dt}=f\left(O,E\right),$$

(2b) $$\frac{dE}{dt}=g\left(O,E\right),$$

where environmental change (*g*) is now governed not only by the environment itself but also by the organisms inhabiting it. This formulation is taken to emphasize that organisms are not passive objects being moulded into a pre‐existing environment, but instead play an active role in both choosing (Morris 2011) and modifying the environment they inhabit. In Lewontin's words: ‘“Organisms do not adapt to their environments; they construct them out of the bits and pieces of the external world.” This is the basic idea of “Niche Construction Theory” (NCT), a framework presented by its proponents as an extension of evolutionary theory (Odling‐Smee et al. 2013). Niche Construction (NC) is defined as “the process whereby organisms, through their activities and choices, modify their own and each other's niches” (Laland and O'Brien 2011). In this context, a niche is defined as “the sum of all the natural selection pressures to which the population is exposed” (Odling‐Smee et al. 2013). This evolutionary niche concept is not to be confused with an ecological niche, which describes conditions under which a population can exhibit positive growth (Hutchinson 1957). NCT emphasizes that coevolution of organisms and their environments can change the flow of energy and matter through ecosystems, often creating new resources used by the same and by other species (Laland and O'Brien 2011). By virtue of its broad definition—which includes effects that can be directed or undirected; positive or negative; mediated by alterations of the habitat or of the organisms themselves—NC is seen as “a very general process, exhibited by all living organisms” (Odling‐Smee et al. 2013). At the same time, NCT does not treat all these effects as equally important, emphasizing “how selection pressures are changed by evolving organisms in *nonrandom* or *directional*, ways” (Scott‐Phillips et al. 2014; emphasis added). The challenge which NCT poses for Williams’ asymmetry has been characterized by Laland et al. (2009, p. 197) as follows:

*“In standard models, leaving aside complications, such as co‐evolution and habitat selection, adaptation is a process by which natural selection shapes organisms to fit pre‐existing environmental “templates.” The causal arrow points in one direction only: environments are the source of selection, and they determine the features of living creatures. According to Williams (* 1992 *, p. 484): “Adaptation is always asymmetrical; organisms adapt to their environment, never vice versa.” This stance is based on some metaphysical assumptions that have underpinned evolutionary thought since the Modern Synthesis. In our view, these assumptions are the source of the conceptual barriers that impede further progress in evolutionary biology.”*

In contrast to the impression conveyed by Equation (1b), we think no plausible claim can be made that evolutionists prior to Lewontin (1983) had overlooked that organisms influence their selective environment. Darwin (1881) devoted a whole book to describing how earthworms are adapted to live in an environment that they have shaped through their own activities. Proponents of NCT acknowledge that NC is indeed a long‐established biological phenomenon (albeit not under that name), but nevertheless claim that NC has not been recognized as a “causal evolutionary process” (Feldman et al. 2017) in SET. Critics have rejected this claim, contending that eco‐evolutionary feedback (i.e., organisms influencing their environment and vice versa) has long been a standard ingredient of evolutionary explanations (Gupta et al. 2017a, b). Moreover, in the evolutionary literature, prominent claims of exclusive unidirectional causality appear to be lacking. Laland et al. (2011) attributed such a claim to Mayr (1961), but see Dickins and Barton (2013). We conclude from this, in agreement with Gupta et al. (2017b), that Equation (1) gives a misleading characterization of SET.

With the alleged misconception of Equation (1) out of the way, we now turn to a more substantive issue. NTC proponents have claimed that “NC provides a second evolutionary route to establishing the adaptive fit, or match, between organism and environment” (Laland and O'Brien 2011), amounting to “an alternative to the standard account of how the adaptive complementarity between organisms and their environments arises” (Scott‐Phillips et al. 2014). We regard this as the central point of NCT, and the reason it has generated so much controversy.

As our opening quote from Williams shows, Lewontin (1983) was right about one thing: there is indeed a fundamental asymmetry in how SET characterizes the relationship between organisms and their environments. This is seen by some as a crucial flaw of SET (Bickerton 2009; Smith 2011; Zeder 2012). But the superficial symmetry of Equation (2a & b) hides a fundamental difference between them; only Equation (2a) focusses on natural selection. In other words, the asymmetry in SET is specifically concerned with *biological adaptation*, rather than with the mere presence or absence of arbitrary cause‐effect relationships. Adaptive evolution is special in being characterized by *directed* modifications toward phenotypes that increase individual fitness. This directedness (or “quasi‐directedness,” if one wishes to emphasize the process's lack of foresight) arises because, in Darwin's (1859) words, “by the accumulation of innumerable slight variations, each good for the individual possessor,” natural selection channels undirected variation into trends of cumulative improvement. The importance of this Darwinian tendency for cumulative improvement can hardly be overstated, as it remains our only scientific explanation for complex adaptive design in nature. Indeed, the similarities between organisms and human‐made artifacts which were historically interpreted as evidence for a supernatural creator (Paley 1802) can be understood as a direct consequence of the (quasi‐) directedness of the underlying processes. Note that, although other species in the environment also evolve, this will not manifest in Equation (2b) as directed toward improving the focal species’ adaptedness.

Was Lewontin—who published his equations repeatedly (Lewontin 2003, 2000)—unaware of the distinction between directed and undirected effects? No, he took a keen interest in this distinction but questioned the justification for regarding adaptation as directed. Lewontin (1983) granted that pre‐existing ecological problems (i.e., selection pressures that persist throughout a species’ evolution)—if they did exist—could explain the evolution of complex well‐adjusted design. It is easy to see why: given consistent selection (and suitable variation) for, say, the best foragers, appropriate modification seems only a matter of time. Yet Lewontin realized that real ecological problems are *not* predetermined in this way: even in the idealized case where almost the entire world is taken as fixed, ecological problems may change as the species changes and/or modifies its environment. This insight may challenge the traditional view of adaptive evolution, if Lewontin (1983) was correct in claiming that: “the classical Darwinian theory of adaptation, […] depends absolutely on the problem preexisting the solution.”

Lewontin was right that ecological problems are not predetermined, even in the partial sense implied by Equation (1b). He did not, however, make a strong case for why this should overturn our conceptual understanding of evolution. Ariew and Lewontin (2004) used a lock‐and‐key metaphor to argue that the word “fit” (“fittest,” “fitness”), as an extension of its everyday English meaning, implies matching an object to a pre‐existent and independently determined pattern. This argument suffers from the weakness that there is no direct logical link between a word's origin and the concept thereby expressed (Heller et al. 1984). But even on its own terms the argument fails: by switching to a shoe‐and‐foot metaphor, we can see that the everyday meaning of “fit” does not imply unidirectional causality. Like a lock, a shoe has a predetermined form that determines which object will fit in it. But then, during “breaking in,” the foot affects the shoe's shape to improve the fit.

From a theoretical perspective one might expect that sufficiently drastic and erratic environmental change—whether caused by organisms or not—could stop any trends for cumulative improvement in their tracks. Yet this concern is defused by the empirical observation that organisms exhibit “that perfection of structure and coadaptation which most justly excites our admiration” (Darwin 1859, p. 3), indicating that cumulative improvement has taken place. Thus, while environmental change may lead to “adaptive lag” (Burger and Lynch 1995; Matuszewski et al. 2015, 2014), we have no reason to think that it fundamentally alters the logic of adaptive evolution. Indeed, because the functioning of different traits may be more or less sensitive to environmental parameters, cumulative improvement may continue even through periods of drastic environmental change. For example, for a predatory species, visual acuity and fast reflexes may be consistently favored even as temperatures change.

Note that this reasoning stands and falls with the existence of an abstract criterion of good phenotypic design that can guide evolution, even as species and their environment change. Without a criterion for what counts as “good,” there can be no recognition of cumulative improvement. But what is this criterion? Lewontin considered this question repeatedly (Lewontin 1978, 1983, 2003; Ariew and Lewontin 2004), but found no satisfactory answer. He considered two candidate classes of answers, namely “reproductive” versus “kinetic” fitness concepts.

“Reproductive fitness,” measured as number of offspring (i.e., lifetime reproductive success; LRS), is intuitively appealing because, other things equal, genes (and hence heritable traits) associated with higher LRS must come to predominate in a population. The problem with this argument, however, is that “other things” are rarely equal. For example, in growing or shrinking populations, the timing of reproduction matters for which variants will increase in frequency (Charlesworth and Giesel 1972). This led Lewontin (2003) to reject LRS as a phenotypic criterion of what is favored.

“Kinetic fitness” (Lewontin 1983), also described as “fitness as an outcome” (Ariew and Lewontin 2004) refers to approaches which assign the highest fitness to variants observed (or calculated) to increase in frequency, thereby making fitness an infallible “predictor” of change. However, since such concepts merely reaffirm the observed, they neither explain why change occurs (Lewontin 2003; Ariew and Lewontin 2004), nor provide criteria that could be subject to cumulative improvement. For example, because offspring do not generally inherit the (multilocus) genotype of either of their parents, there is no simple link between a genotype's performance and its frequency. Similarly, because phenotypic variation in sexually reproducing species does not generally come in the form of discrete types, a phenotype's performance cannot be characterized as an increase in type frequency.

Ariew and Lewontin (2004) note—with justification, in our view—that difficulties with the definition of fitness are commonly obscured by switching between concepts. Biologists may think that cumulative improvement explains complex adaptive design, yet, when confronted with the limitations of LRS, turn to “kinetic” fitness concepts that provide no criterion of improvement. At the same time, by using suitable simplifying assumptions, theoreticians can sidestep the need for an explicit improvement criterion. For example, when modeling phenotypic evolution as if it was governed by one gene at a time (i.e., “adaptive dynamics” as applied to sexual organisms (Geritz et al. 1998), the focal gene's growth rate can serve to indicate the direction of evolution.

Do the above considerations force us to fall back on the crude picture of causality of Equations (1–2)? No, because they do not exhaust the possibilities. Williams (1966b), drawing on Fisher (1930), identified reproductive value (RV) as the currency in which costs and benefits of adaptive traits should be measured (Houston and McNamara 1999; Crewe et al. 2018). Accordingly, an evolutionarily meaningful measure of LRS must weight offspring by RV, that is, by each offspring's expected contribution to the future gene pool. For example, if instead of producing one typical offspring, a parent could produce *X* offspring whose per‐capita contribution to the future gene pool was *Y* times higher (such that X*Y  > 1; e.g., due to improved survival and/or reproduction), then by choosing the latter option the parent will increase its own contribution to the future gene pool. Grafen (1999) showed that similar considerations can take care of changes in population size. Because one individual among few will make a larger proportional contribution to the gene pool than one among many, each offspring's RV is inversely related to population size at its time of birth. Thus, while the timing of reproduction matters insofar as it places offspring in different contexts, natural selection nevertheless favors individuals that maximize their RV‐weighted LRS (Grafen 1999, 2014; Crewe et al. 2018).

It is worth saying that purely phenotype‐based predictions about evolution are necessarily heuristic in nature. Hence, in the statement of Ariew and Lewontin (2004) that “a type's Darwinian ‘fitness’ to the environment implies a single ordinal scalar *which will predict the relative increase or decrease of the type*” [emphasis added], the highlighted part is not strictly true. Depending on genetic details, natural selection will not always favor phenotypic improvement in the short term (Hammerstein 1996). For example, high‐performing allele combinations can be broken up by segregation; and various kinds of “rogue genes” (e.g., segregation distorters) can spread at a cost to organisms’ reproductive success. In the long run, however—over timespans needed for substantial trends of cumulative improvement—these caveats may play only a minor role. Since such trends probably involve contributions from numerous genes, they should come to reflect the majority interest of the “parliament of genes” (Leigh 1971), which is to build organisms that achieve high fitness, or, more generally, inclusive fitness (Hamilton 1964; Fromhage and Jennions 2019). While the true genetic complexity of long‐term trends in adaptive evolution may be beyond mathematical description, simulations suggest that the parliament of genes’ majority interest will tend to prevail under realistic genetic assumptions (Scott and West 2019; Garcia‐Costoya and Fromhage 2021).

It is undisputed that organisms sometimes modify their habitat in a directed manner resembling human construction work. A popular example is dam building by beavers, and even authors who are otherwise critical of NCT have considered the term NC apt for such cases (Dawkins 2004). It does not follow, however, that such directed modifications present a challenge to Williams’ asymmetry. This is so because Williams’ asymmetry is concerned with a *particular kind* of directedness, namely toward adaptive traits. In the words of Williams (1966), “an organic adaptation would be a mechanism designed to promote the success of an individual organism, as measured by the extent to which it contributes genes to later generations of the population of which it is a member. It has the individual's inclusive fitness (Hamilton 1964) as its goal.” In other words, biological adaptedness manifests in organisms expressing traits whose effect is to elevate their individual bearers’ inclusive fitness above what they would attain otherwise. For example, if dam building in beavers is adaptive, then an individual beaver who builds a dam will thereby attain higher inclusive fitness than if it had not built the dam. Note that this positive effect on the builder's inclusive fitness can only manifest if the dam was not there to begin with. So, in this example, dam‐building opportunities (e.g., un‐dammed creeks), rather than completed dams, are the habitat feature which improves the fit between beavers and their environment.

Note that the relevant concept of environment here is not identical with the physical habitat, but instead corresponds to the totality of conditions (including the beavers’ current traits) that determine the causal mapping between phenotypes and fitness (Fig. 1). If we saturated the landscape with dams and lakes by human intervention, this would presumably reduce the organism‐environment fit in such a way as to select against beavers’ dam‐building ability in the long run (at least if this ability has costs). This makes it far from obvious whether, as is often assumed, dam‐building improves the organism–environment fit in an evolutionarily relevant sense.

**Figure 1:**
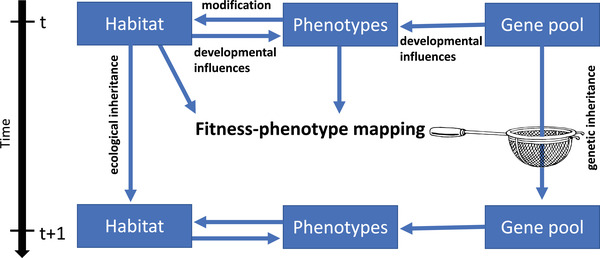
Schematic representation of adaptive evolution and other causal forces. Organisms can influence their habitat and vice versa, leading to ecoevolutionary feedback. In contrast to other causal forces, however, adaptive evolution shapes organisms toward matching their environment, in the sense of exhibiting traits that confer high inclusive fitness in that environment. On this view, the environment to which organisms adapt is not identical with the physical habitat, but instead corresponds to the totality of conditions (including the focal species’ current traits) that determine the mapping of fitness on phenotypes. This mapping (here symbolised by a sieve) biases gene propagation toward genes whose effect is to build better‐adapted organisms. The (quasi‐) directionality inherent in this process enables trends of cumulative improvement, which underlie much of evolution's creative potential.

For the sake of argument, let us invent a best‐case scenario where a habitat modification unquestionably improves the organism–environment fit. Imagine a hypothetical world, where beavers live in a landscape without large water bodies, yet where, for some unrelated reason (say, genetic drift) they still evolve flat tails and webbed feet. If these beavers then follow a spontaneous impulse to take up dam building (without being selectively favored to do so—this detail matters), then the resulting lakes will render the beavers’ aquatic traits more useful than before for gaining fitness. This improves the organism–environment fit through a modification of the environment rather than of the organisms, but not in a directed fashion but by coincidence. Compare this with an otherwise identical case, where beavers are *selected* to take up dam building. In this case, because the beavers’ pre‐existing aquatic traits contribute to the fitness‐phenotype mapping (Fig. 1), they should make dam building more beneficial than it would otherwise be. Like any other trait, they can be considered as part of the background conditions that select for dam building in the usual Darwinian way. Thus, the directed process by which aquatic traits come to qualify as fitness‐increasing is just ordinary adaptative evolution acting on another trait. The lesson we draw from this thought experiment is that, even if we grant the (uncertain) claim that real beaver dams increase the organism–environment fit for beavers, no directed process other than organismal adaptation is needed to explain how this has come to pass over evolutionary time.

Let us briefly consider another example. A cheetah possesses specialised traits for hunting (e.g., long legs, light body; Hudson et al. 2011) that make it well‐fitted to an environment with fast prey. When making a kill, a cheetah certainly changes its habitat in a directed manner: it converts a potential prey into a lump of meat. Yet although hunting is adaptive for cheetahs, it does not follow that its outcome (the presence of meat) improves the organism–environment fit in the evolutionarily relevant sense. Indeed, if we would regularly provide cheetahs with killed prey, then this intervention would render their hunting adaptations useless, decreasing the organism–environment fit.

There are, to be sure, other senses in which beaver dams, killed prey, and many other habitat modifications might be considered to improve the organism–environment fit, for example, aesthetically or by increasing population size or viability. The latter possibilities are readily evoked by the term “Niche” through its association with the concept of “Ecological Niche,” which is concerned with the thriving of populations (Hutchinson 1957; Holt 2009). We stress, however, that these other senses are strictly beside the point when considering biological adaptation.

We conclude that habitat modifications through the directed activities of organisms do not systematically tend to increase the adaptive fit between organisms and their environment. And even when they occasionally do, no alternative directed force other than organismal adaptation is needed to explain how this fit became established over evolutionary time.

We give the last word to Williams (1992): “With a more parochial focus on our current and immediate environment, it may appear that conditions are eminently suitable for ourselves and other organisms. This impression stems from failure to appreciate how completely one‐sided adaptation is, and what it can be expected to accomplish. Living organisms are elaborately adapted to their particular ways of life in the environments in which they evolved. There is no evidence for any other kind of adaptation.”

Associate Editor: M. Kopp

Handling Editor: T. Chapman
